# Complete Genome Sequence of Spiroplasma floricola 23-6^T^ (ATCC 29989), a Bacterium Isolated from a Tulip Tree (Liriodendron tulipifera L.)

**DOI:** 10.1128/genomeA.00302-18

**Published:** 2018-04-19

**Authors:** Yi-Ming Tsai, Pei-Shan Wu, Wen-Sui Lo, Chih-Horng Kuo

**Affiliations:** a Institute of Plant and Microbial Biology, Academia Sinica, Taipei, Taiwan

## Abstract

Spiroplasma floricola 23-6^T^ (ATCC 29989) was isolated from the flower surface of a tulip tree (Liriodendron tulipifera L.). Here, we report the complete genome sequence of this bacterium to facilitate the investigation of its biology and the comparative genomics among Spiroplasma species.

## GENOME ANNOUNCEMENT

The strain Spiroplasma floricola 23-6^T^ was isolated from a tulip tree (Liriodendron tulipifera L.) flower collected in the state of Maryland in the United States (1–3). This bacterium was shown to be pathogenic to larvae of the greater wax moth (Galleria mellonella) in an artificial infection experiment (4). To facilitate future investigation of the biology of this bacterium, as well as to improve the taxon sampling of available Spiroplasma sequences for comparative genomics and evolutionary studies (5), we determined its complete genome sequence.

The strain was acquired from the American Type Culture Collection (catalog number ATCC 29989). The freeze-dried sample was processed according to the manufacturer’s instruction and cultured in M1D medium (6) prior to DNA extraction using the Wizard Genomic DNA purification kit (Promega, USA). PCR and Sanger sequencing were performed to verify that the 16S rRNA gene sequence matched the reference record (GenBank accession number AY189131) (7).

The procedures for genome sequencing, assembly, and annotation were based on those described in our previous studies (8–19). Briefly, the Illumina platform was used to generate raw reads from one paired-end library (∼283-bp insert, ∼416-fold coverage) and one mate-pair library (∼4,000-bp insert, ∼476-fold coverage). The initial *de novo* assembly was performed using ALLPATHS-LG release 52188 (20). Subsequently, PAGIT version 1 (21) was used to assist an iterative process for improving the assembly. For each iteration, the raw reads were mapped to the assembly using BWA version 0.7.12 (22), programmatically checked using the mpileup program in the SAMtools package version 1.2 (23), and visually inspected using IGV version 2.3.57 (24). Polymorphic sites and gaps were corrected based on the mapped reads. The process was repeated until the complete genome sequence was obtained. The programs RNAmmer (25), tRNAscan-SE (26), and Prodigal (27) were used for gene prediction. The gene names and product descriptions were first annotated based on the homologous genes in other Spiroplasma genomes (8–19) as identified by OrthoMCL (28). Subsequent manual curation was based on the information obtain from the BlastKOALA tool (29) and BLASTP (30) searches against the NCBI nonredundant database (31). Putative clustered regularly interspaced short palindromic repeats (CRISPRs) were identified using CRISPRFinder (32).

The complete genome sequence of Spiroplasma floricola 23-6^T^ consists of one circular chromosome that is 1,284,130 bp in size with 24.3% G+C content; no plasmid was found. The first version of annotation includes one set of 16S-23S-5S rRNA genes, 29 tRNA genes (covering all 20 amino acids), 1,122 protein-coding genes, 8 pseudogenes, and 1 CRISPR locus (chromosomal positions 565,140 to 565,547, containing five spacers).

### Accession number(s).

The complete genome sequence of Spiroplasma floricola 23-6^T^ has been deposited at DDBJ/EMBL/GenBank under the accession number CP025057.
